# Supplementary material for: Olfactory combinatorial coding supports risk–reward decision-making in Caenorhabditis elegans
Source: eLife. 2026 Oct 22;14:RP104828. doi: 10.7554/eLife.104828 (PMC13690342; doi:10.7554/eLife.104828)
Supplement: Peer review history

# Editor report: eLife Assessment

**DOI:** 10.7554/eLife.104828.4.sa0

**Authors:** Andrew J King

**Affiliations:** University of Oxford United Kingdom

**Keywords:** Convincing, Important

This **important** study shows that an odorant that is typically thought of as a repellent actually activates both attractant and repellent olfactory neurons in *Caenorhabditis elegans*. **Convincing** evidence is provided that nematode worms can integrate signals in different sensory pathways to drive different behavioral responses to the same cue. These findings will be of interest to scientists interested in combinatorial coding in sensory systems.

# Referee report: Reviewer #1 (Public review)

**DOI:** 10.7554/eLife.104828.4.sa1

[Editors' note: this version has been assessed by the Reviewing Editor without further input from the original reviewers.]

The authors investigated the response of worms to the odorant 1-octanol (1-oct) using a combination of microfluidics-based behavioral analysis and whole-network calcium imaging. They hypothesized that 1-oct may be encoded through two simultaneous, opposing afferent pathways: a repulsive pathway driven by ASH, and an attractive pathway driven by AWC. And the ultimate chemotactic outcome is likely determined by the balance between these two pathways.

It is not surprising that 1-octanol is encoded as attractive at low concentrations and repulsive at higher concentrations. However, the novel aspect of this study is the discovery of the combinatorial coding of 1-oct in the periphery, where it serves as both an attractant and a repellent. Furthermore, the study uses this dual encoding as a model to explore the neural basis of sensory-driven behaviors at a whole-network scale in this organism. The basic conclusions of this study are well supported by the behavioral and imaging experiments, though there are certain aspects of the manuscript that would benefit from further clarification.

A key issue is that several previous studies have demonstrated a combinatorial and concentration-dependent coding of odorant sensing in the nematode peripheral nervous system. Specifically, ASH and AWC are the primary receptors for repellent and attractive responses, respectively. However, other neurons such as AWB, AWA, and ADL are also involved in the coding process. These neurons likely communicate with different interneurons to contribute to 1-oct-induced outputs. The authors' conclusion that loss of tax-4 reduces attractive responses and that osm-9 mutants reduce repulsive responses is not entirely convincing. TAX-4 is required for both AWC (an attractive neuron) and AWB (a repulsive neuron), and osm-9 is essential for ASH, ADL, and AWA (attraction-associated). Therefore, the observed effects on the attractive and repulsive responses could be more complex. Additionally, the interpretation of results involving the use of IAA to reduce the contribution of AWC at lower concentrations lacks clarity.

The authors did not observe any increased correlation between motor command interneurons and sensory neurons, which is consistent with the absence of a consistent relationship between state transitions and 1-oct application. Furthermore, they did not observe significant entrainment of AIB activity with the 2.2 mM 1-oct application. This might be due to the animals being anesthetized with 1 mM tetramisole hydrochloride, which could affect neural activity and/or feedback from locomotion.

# Referee report: Reviewer #2 (Public review)

**DOI:** 10.7554/eLife.104828.4.sa2

Summary:

The authors used whole-network imaging to identify sensory neurons that responded to the repellent 1-octanol. While several olfactory neurons responded to the initial onset of odor pulses, two neurons consistently responded to all the pulses, ASH and AWC. ASH typically activates in response to repellents, and AWC typically activates in response to the removal of attractants. However, in this case, AWC activated in response to the removal of 1-octanol, which was unexpected because 1-octanol is a harmful repellent to the worm. The authors further investigated this phenomenon by testing different concentrations of 1-octanol in a chemotaxis assay and found that at lower (less harmful) concentrations the odor is actually an attractant, but becomes repulsive at higher concentrations. The amplitude of the ASH response appeared to be modulated by concentration, but this was not true for AWC. The authors propose a model where the behavioral response of the worm is the result of integrating these two opposing drives, where repulsion is a result of the increased ASH activity over-riding the positive drive from AWC. The authors further tested this theory by testing mutants that ablated the AWC response (tax-4 or AWC::HisCl) or ASH response (osm-9 or ASH::HisCl). The chemo-silencing (HisCl) and tax-4 experiments were consistent with their hypothesis, while the osm-9 mutation had a limited impact on chemotaxis behavior, highlighting the potential role of osm-9-independent signaling in ASH in response to 1-octanol. While the interneuron(s) that integrate these signals to influence behavior were not identified, the authors did find that increasing concentrations of 1-octanol did increase the likelihood of AVA activity, a neuron which drives reversals (and hence, behavioral repulsion).

Strengths:

This was simple and elegant work that identified specific neurons of interest which generated a hypothesis, which was further tested with mutants that altered neuronal activity. The authors performed both neuronal imaging and behavioral experiments to verify their claims.

Weaknesses:

The authors note that other sensory neurons likely contribute to 1-octanol chemotaxis. Given the NeuroPAL data, it would have been nice to identify these other neurons as well. However, the reviewer is aware that this is tangential to the primary focus of this study.

# Referee report: Reviewer #3 (Public review)

**DOI:** 10.7554/eLife.104828.4.sa3

Summary:

This work describes how two chemosensory neurons in *C. elegans* drive opposite behaviors in response to a volatile cue. Because they have different concentration dependencies, this leads to different behavioral responses (attraction at low concentration and repulsion at high concentration). It has been known that many odorants that are attractive at low concentrations are aversive at high concentrations, and the implicated neurons (at least AWC for attraction and ASH for repulsion) have been well established. Nonetheless, by studying behavior and neural responses in a common context (odor pulses, as opposed to gradients) this provides a clear picture of how these sensory neurons may guide the dose dependent response by separately modulating odor entry and odor exit behaviors.

Strengths:

(1) This work provides good evidence that worms are attracted to low concentrations and repelled by high concentrations of 1-oct. Calcium imaging also makes it clear that dose-dependence of this response is stronger for ASH than AWC.

(2) This work presents calcium imaging and behavior with the same stimulus (sudden pulses in volatile odor concentration), while previous studies often focus on using neuronal responses to pulses to understand navigation of gentle gradients.

Weaknesses:

(1) As a whole it is not clear precisely how important AWC is (compared to other cells) for the attractive response (as the authors correctly acknowledge).

(2) The evidence that AIB minus AVA contains relevant information is weak. It appears the entrainment index in Fig. 6H for AIB-AVA could easily be explained by the negative entrainment between AVA and the stimulus (along with no effect or role for AIB). This is suggested by the similar p-values and similar distribution of random EIs (stretched and mirrored) between the first and last rows of this figure.

# Author comment: Author response

**DOI:** 10.7554/eLife.104828.4.sa4

**Authors:** Md Zubayer Hossain Saad, William G Ryan, Chelyan A Edwards, Benjamin N Szymanski, Lana Awa, Jenna Kaake, Alexander Martin, Aryan R Marri, Lilian G Jerow, Robert McCullumsmith, Bruce A Bamber

**Affiliations:** University of Toledo Toledo United States; University of Toledo Medical Center Toledo United States; University of Toledo Toledo United States; University of Toledo Toledo United States; The University of Toledo Toledo United States; The University of Toledo Toledo United States; The University of Toledo Toledo United States; University of Toledo Toledo United States; University of Cincinnati Cincinnati United States; University of Toledo Medical Center Toledo United States; University of Toledo Toledo United States

The following is the authors’ response to the previous reviews

**Public Reviews:**

**Reviewer #1 (Public review):**

The authors investigated the response of worms to the odorant 1-octanol (1-oct) using a combination of microfluidics-based behavioral analysis and whole-network calcium imaging. They hypothesized that 1-oct may be encoded through two simultaneous, opposing afferent pathways: a repulsive pathway driven by ASH, and an attractive pathway driven by AWC. And the ultimate chemotactic outcome is likely determined by the balance between these two pathways.

It is not surprising that 1-octanol is encoded as attractive at low concentrations and repulsive at higher concentrations. However, the novel aspect of this study is the discovery of the combinatorial coding of 1-oct in the periphery, where it serves as both an attractant and a repellent. Furthermore, the study uses this dual encoding as a model to explore the neural basis of sensory-driven behaviors at a whole-network scale in this organism. The basic conclusions of this study are well supported by the behavioral and imaging experiments, though there are certain aspects of the manuscript that would benefit from further clarification.

A key issue is that several previous studies have demonstrated a combinatorial and concentration-dependent coding of odorant sensing in the nematode peripheral nervous system. Specifically, ASH and AWC are the primary receptors for repellent and attractive responses, respectively. However, other neurons such as AWB, AWA, and ADL are also involved in the coding process. These neurons likely communicate with different interneurons to contribute to 1-oct-induced outputs. The authors' conclusion that loss of tax-4 reduces attractive responses and that osm-9 mutants reduce repulsive responses is not entirely convincing. TAX-4 is required for both AWC (an attractive neuron) and AWB (a repulsive neuron), and osm-9 is essential for ASH, ADL, and AWA (attraction-associated). Therefore, the observed effects on the attractive and repulsive responses could be more complex. Additionally, the interpretation of results involving the use of IAA to reduce the contribution of AWC at lower concentrations lacks clarity.

The authors did not observe any increased correlation between motor command interneurons and sensory neurons, which is consistent with the absence of a consistent relationship between state transitions and 1-oct application. Furthermore, they did not observe significant entrainment of AIB activity with the 2.2 mM 1-oct application. This might be due to the animals being anesthetized with 1 mM tetramisole hydrochloride, which could affect neural activity and/or feedback from locomotion.

Comments on revisions:

The authors have addressed all my previously raised concerns.

**Reviewer #2 (Public review):**

Summary:

The authors used whole-network imaging to identify sensory neurons that responded to the repellent 1-octanol. While several olfactory neurons responded to the initial onset of odor pulses, two neurons consistently responded to all the pulses, ASH and AWC. ASH typically activates in response to repellents, and AWC typically activates in response to the removal of attractants. However in this case, AWC activated in response to the removal of 1-octanol, which was unexpected because 1-octanol is a harmful repellent to the worm. The authors further investigated this phenomenon by testing different concentrations of 1-octanol in a chemotaxis assay, and found that at lower (less harmful) concentrations the odor is actually an attractant, but becomes repulsive at higher concentrations. The amplitude of the ASH response appeared to be modulated by concentration, but this was not true for AWC. The authors propose a model where the behavioral response of the worm is the result of integrating these two opposing drives, where repulsion is a result of the increased ASH activity over-riding the positive drive from AWC. The authors further tested this theory by testing mutants that ablated the AWC response (tax-4 or AWC::HisCl) or ASH response (osm-9 or ASH::HisCl). The chemo-silencing (HisCl) and tax-4 experiments were consistent with their hypothesis, while the osm-9 mutation had a limited impact on chemotaxis behavior, highlighting the potential role of osm-9-independent signaling in ASH in response to 1-octanol. While the interneuron(s) that integrate these signals to influence behavior were not identified, the authors did find that increasing concentrations of 1-octanol did increase the likelihood of AVA activity, a neuron which drives reversals (and hence, behavioral repulsion).

Strengths:

This was simple and elegant work that identified specific neurons of interest which generated a hypothesis, which was further tested with mutants that altered neuronal activity. The authors performed both neuronal imaging and behavioral experiments to verify their claims.

Weaknesses:

The authors note that other sensory neurons likely contribute to 1-octanol chemotaxis. Given the NeuroPAL data, it would have been nice to identify these other neurons as well. However, the reviewer is aware that this is tangential to the primary focus of this study.

**Reviewer #3 (Public review):**

Summary:

This work describes how two chemosensory neurons in *C. elegans* drive opposite behaviors in response to a volatile cue. Because they have different concentration dependencies, this leads to different behavioral responses (attraction at low concentration and repulsion at high concentration). It has been known that many odorants that are attractive at low concentrations are aversive at high concentrations, and the implicated neurons (at least AWC for attraction and ASH for repulsion) have been well established. None the less, by studying behavior and neural responses in a common context (odor pulses, as opposed to gradients) this provides a clear picture of how these sensory neurons may guide the dose dependent response by separately modulating odor entry and odor exit behaviors.

Strengths:

(1) This work provides good evidence that worms are attracted to low concentrations and repelled by high concentrations of 1-oct. Calcium imaging also makes it clear that dose-dependence of this response is stronger for ASH than AWC.

(2) This work presents calcium imaging and behavior with the same stimulus (sudden pulses in volatile odor concentration), while previous studies often focus on using neuronal responses to pulses to understand navigation of gentle gradients.

Weaknesses:

(1) As a whole it is not clear precisely how important AWC is (compared to other cells) for the attractive response (as the authors correctly acknowledge).

(2) The evidence that AIB minus AVA contains relevant information is weak. It appears the entrainment index in Fig. 6H for AIB-AVA could easily be explained by the negative entrainment between AVA and the stimulus (along with no effect or role for AIB). This is suggested by the similar p-values and similar distribution of random EIs (stretched and mirrored) between the first and last rows of this figure.

(3) The model in Figure 7 would be strengthened if it was demonstrated that IAA is attractive when worms are saturated in a 1/10^4 concentration. Panel 7G (and ref. 39) indicate that 10^-4 IAA activates ASH, which would suggest a different explanation for the change from attraction to repulsion in 7C.

It was previously published that 1x10^-4 IAA is attractive in a similar microfluidics context (Albrecht and Bargmann), and we have confirmed its attractiveness in our experimental setup (not shown). Specifically, worms accumulate in zones of the arena where 1x10^-4 IAA is present: they are attracted upon first encounter and maintain attraction after prolonged exposure. A sentence to this effect has been added to Results (line 402).

**Recommendations for the authors:**

**Reviewer #2 (Recommendations for the authors):**

This looks great! My only small recommendation is to include the 1-octanol concentration used (2.2 mM) in Fig 4 F,G so that the reader can easily compare these data to Fig 4 D,E. You can do this in the figure or legend, though in the figure would be preferable (as you did for Fig 5 and Fig 6).

[1-oct] added to Fig. 4D, E

**Reviewer #3 (Recommendations for the authors):**

(1) The AWC traces in Fig. 3C do not match, despite them being from the same animal. There appear to be too many ASH traces. NeuroPAL data/traces from other animals were not available on Zenodo when this review was prepared. This should be fixed.

Single-animal traces added to figure so reader can see the relationship in the single animal shown and across the full dataset. The averaged ASH/AWC traces are retained in the updated figure because this observation is central to the main hypothesis, so it is important to demonstrate it is a consistent phenomenon. Legend modified (lines 1016-17), ‘consistent’ added to Results (line 147). Data will be uploaded to Zenodo upon finalization of the Version of Record

(2) The normalization of traces in Fig. 4 is not clear (panel A is not min-max, as panel B appears to be). This normalization may be critical to the interpretation that ASH responds dose-dependently. The code linked on Zenodo was not available when this review was prepared. This should be fixed.

The reviewer correctly points out a slight error in normalization of the individual worm traces for the ascending concentration series (4A, left panel; the maximal value shown was slightly under 1.0). This is now corrected, interpretation unchanged.

(3) Chemogenetic data (4F,G and 5D) should specify the promoters used, as they are not ASH and AWC-specific.

Figures 4F, G and 5D altered to specify the promoters used, as requested. As before, the full range of neurons in which these promoters are active is stated in the Discussion
